# The Cycle of Life

**Published:** 1895-08-10

**Authors:** 


### The Cycle of Life.

Among the problems in biology which at the present time force themselves most prominently upon the attention of the inquiring student of nature, none are more interesting than those which are concerned with the very borderland between the organic and the inorganic, with the processes by which the dead and simple elements of the world around are enabled to take upon themselves, or become endued with, the complex constitution and the wonderful attributes of life.

That animals cannot live on the crude elements we all know, and even our school children are now initiated into the wonderful mystery, the cycle of life, the interdependence of vegetables and animals, and are taught how, by the activity of the chlorophyll apparatus of the green plant, the sun's rays are made to build up from the water and carbonic acid in the atmosphere an infinite series of carbohydrates charged with potential energy, and how by the processes of assimilation and metabolism in the animal body these carbohydrates, when taken as food, are made to discharge their energy in the form of heat and bodily activity, the dead hydrogen being returned as water to the earth and the dead carbon as carbonic acid to the atmosphere. This cycle of life, this apparent balance between the functions of the vegetable and the animal sides of nature, has long been a favourite theme with teachers of elementary science, and has undoubtedly been for many students the germ of truer ideas, the basis of larger conceptions of nature's works than were possible to their forefathers.

But the schoolmaster has had to let nitrogen alone. Undoubtedly this element also has its circulation. We do not pass our lives bathed in an atmosphere of nitrogen all for nothing. We know that nitrogen is absolutely essential to living matter; we know that living matter is constantly parting with compounds containing it, and that these compounds are being continually washed away into the deeper recesses of the ground far from the reach of the roots of plants. Whence, then, is it renewed, and by what mechanism is the dead nitrogen of the atmosphere combined with organic carbohydrates and turned into living protoplasm? This is a question round which controversy has long raged, and it is only of recent years that it has been possible to formulate anything like a complete hypothesis even as to the source from which the nitrogenous constituents of organic substances are derived.

In an interesting article by Professor H. Marshall Ward, F.R.S., which has recently appeared in *Science Progress*, the whole question is reviewed, and it is shown that by the aid and intervention of certain micro-organisms the fixation of free nitrogen by plants is accomplished.

To older observers the soil appeared a dead thing, from which the roots of plants sucked up the water and the salts required for their nutrition. New observers, however, see the matter in a new light; to them the soil itself is living, is teeming with micro-organisms, engaged not merely with processes of decomposition, but occupied in producing those nitrogenous compounds which are as necessary to plant life as are the carbon compounds extracted from the atmosphere.

Whence, however, do these microbes derive the energy by which they can produce such wonderful transformations? The answer is, from the carbohydrates of the plant. We have here an example of that wonderful adaptation of means to an end which is spoken of as symbiosis, the living together of two forms of life not as parasites for mutual destruction, but as associates for mutual help. The green plant with its leaves exposed to the air, and receptive of radiant energy, produces carbohydrates, some of which are used as food by the humble microbes which in turn, working in their mysterious way beneath the ground, use up the energy so derived for the benefit of the plant that gives it, fixing for it free nitrogen, and forming for its use those nitrogenous compounds which are essential for a plant's development. In the case of the leguminosæ especially these nitrogen-fixing microbes grow in and around the roots of the plants, invading the tissues and stimulating them to the formation of nodules in the midst of which they develop. So long as the roots of the plants have plenty of these nodules they are able to obtain nitrogenous compounds even from a nitrogen-free soil, but without the nodules, or, in other words, without the microbes which have the power of fixing the free nitrogen, as is the case in the gramineæ, the plants die of nitrogen starvation. Hence the difference between the cultivation of cereals and leguminous plants, the one exhausting the soil of nitrogen, the other making it actually richer. The story of the fixation of free nitrogen as told in *Science Progress* is full of interest, and is an essential complement to the well-known tale of the fixation of carbon by the leaves of plants

